# RP-HPLC Method for the Determination of Losartan Potassium and Ramipril in Combined Dosage Form

**DOI:** 10.4103/0250-474X.62243

**Published:** 2010

**Authors:** K. Srinivasa Rao, K. Srinivas

**Affiliations:** Roland Institute of Pharmaceutical Sciences, Berhampur-760 010, Orissa, India

**Keywords:** Losartan potassium, ramipril, validation, RP-HPLC

## Abstract

A simple, specific and accurate reverse phase liquid chromatographic method was developed for the simultaneous determination of losartan potassium and ramipril in table dosage forms. A hypersil ODS C18, 4.6×250 mm, 5 μm column in isocratic mode, with mobile phase acetonitrile:methanol:10 mM tetra butyl ammonium hydrogen sulphate in water in the ratio of 30:30:40% v/v/v was used. The flow rate was 1.0 ml/min and effluent was monitored at 210 nm. The retention times of losartan potassium and ramipril were 4.7 and 3.3 min, respectively. The linearity range for losartan potassium and ramipril were in the range of 0.04-100 μg/ml and 0.2-300 μg/ml, respectively. The proposed method was also validated and successfully applied to the estimation of losartan potassium and ramipril in combined tablet formulations.

Losartan potassium (LRT) is chemically 2-butyl-4-chloro-1-[p-(o-1H-tetrazol-5ylphenyl)benzyl]imidazole-5-methanol monopotassium salt. LRT is the first of a unique class of oral antihypertensive agents referred to as angiotensin II receptor antagonists[1]. Ramipril (RAM) is chemically (2*S*, 3*aS*, 6*aS*)-1[(*S*)-*N*-[(*S*)-1-carboxy-3-phenylpropyl]alanyl]octahydrocyclopenta[*b*] pyrrole-2-carboxylic acid-1-ethyl ester. It is an antihypertensive agent. Ramiprilat, the diacid metabolite of ramipril, is a non-sulfhydryl angiotensin converting enzymenone inhibitor[2–3]. RAM is converted to ramiprilat by hepatic cleavage of the ester group.

A literature survey regarding quantitative analysis of these drugs revealed that a few methods[4–13] were reported for the estimation of LRT and RAM individually and only one method is reported so far for the estimation of LRT and RAM in combined dosage forms[14]. This paper described a new RP-HPLC method for the simultaneous estimation of LRT and RAM in combined dosage form using simple mobile phase.

Quantitative HPLC was performed on a binary gradient HPLC with Shimadzu LC10AT and LC10AT VP series HPLC pumps, with a 20 μl injection of sample loop (manual), and SPD 10A VP UV/Vis detector. The output signal was monitored and integrated using Shimadzu Class-VP version 6.12 SP1 software. Hypersil ODS C_18_ (46 mm×25 cm, 5 μm) column was used for the separation. RAM and LRT were gift samples from Sun Pharma Ltd., Vadodara. Formulation A (loram-5, Unichem Ltd., India) containing 5 mg of RAM and 50 mg of LRT was purchased from a local pharmacy. Purified water was prepared using a Millipore Milli-Q (Bedford, MA, USA) water purification system. Acetonitrile of HPLC grade was purchased from Ranbaxy Fine Chemicals Ltd., New Delhi, India. Tetra butyl ammonium hydrogen sulphate was purchased from Hi-media Laboratories Pvt. Ltd., Mumbai, India. Methanol of HPLC grade was purchased for E-Merck, Mumbai, India.

Stock solutions of the RS drugs were prepared by dissolving 25 mg each of RAM and LRT separately in 15 ml acetonitrile contained in 25 ml volumetric flasks and sonicated for 20 min and then the volume was made up to 25 ml with mobile phase. From the standard stock solution, mixed standard solution was prepared to contain 5 μg/ml of RAM, 50 μg/ml of LRT.

Twenty tablets, each containing 5 mg of RAM and 50 mg of LRT were weighed and finally powdered. A quantity of powder equivalent to 0.5 mg of RAM and 5.0 mg of LRT were weighed and transferred to 100 ml volume flask containing 50 ml mobile phase. The mixture was sonicated for 10 min. The volume was made up to 100 ml with mobile phase. The contents were filtered through 0.22 μ membrane filter. An aliquot portion of the filtrate was further diluted to get final concentration of 10 μg/ml of RAM and 100 μg/ml of LRT. This solution was used for the estimation.

The method was validated for accuracy, precision, specificity, limit of detection, limit of quantification and robustness. The accuracy of the method was determined by calculating recoveries of RAM and LRT by method of standard additions. The intra day and inter-day precision study of RAM and LRT was carried out by estimating the corresponding responses 3 times on the same day and on 3 different days of 3 different concentrations of RAM (1, 2, 5 μg/ml) and LRT (1, 3, 6 μg/ml). The repeatability studies were carried out by estimating response of three different concentrations of RAM and LRT for triplicate and results are reported in terms of relative standard deviation (RSD). For specificity study commonly used excipients (starch, microcrystalline cellulose and magnesium stearate) present in selected tablet formulation were spiked into a pre weighed quantity of drugs. The chromatogram was taken by appropriate dilutions and the quantities of drugs were determined. The limit of detection and limit of quantification of the developed method were determined by injecting progressively low concentrations of the standard solutions using the developed RP-HPLC method. Limit of detection was the concentration that yielded signal to noise ratio (S/N) 3:1 and limit of quantification was the concentration that yielded signal to noise ratio (S/N) 10:1. Robustness was done by small changes in the chromatographic conditions and the present method didn't show any significant change when the critical parameters were modified.

Selection of mobile phase was performed based on resolution, asymmetric factors and theoretical plates obtained for both RAM and LRT. The mobile phase acetonitrile: methanol: 10 mM tetrabutylammonium hydrogensulphate (TBHS) in water in the ratio of 30: 30: 40% v/v/v at flow rate of 1.0 ml/min gave sharp peaks with minimum tailing and good resolution for both RAM and LRT. RAM and LRT were eluted at retention times 3.317 and 4.742 min, respectively with symmetric peak shape (fig. 1). UV overlain spectra of RAM and LRT showed that both the drugs are having appreciable absorbance at 210 nm and therefore 210 nm was selected as the detection wavelength in liquid chromatography.

**Fig. 1:**
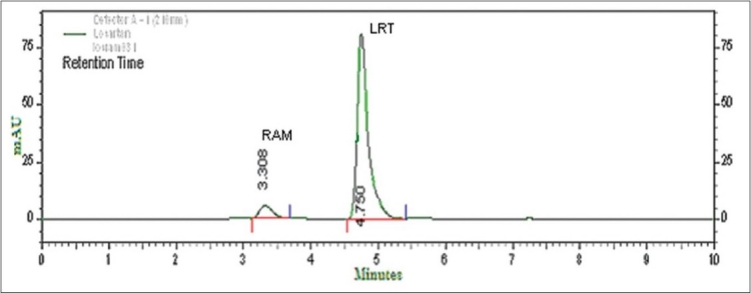
Typical chromatogram of LRT and RAM LRT is losartan potassium; RAM is ramipril

The linearity of the method was determined at different concentration levels from 0.04 to 100 μg/ml for LRT and 0.2 to 300 μg/ml for RAM using RS drug solutions. The total eluting time was less than 10 min. The linearity of this method was evaluated by linear regression analysis, which was calculated by least square method and the data of regression analysis of the calibration curves are shown in Table 1. The mean±standard deviation (SD) for the slope, intercept and correlation coefficient of standard curves (n=6) were calculated. The represented data was shown in Table 2. The % recoveries of RAM and LRT were found to be in the range of 99.52–99.86% and 99.08–99.41%, respectively. The proposed liquid chromatographic method was applied to the determination of RAM and LRT in their combined dosage forms. The results of RAM and LRT were comparable with the corresponding labeled amounts (Table 3).

**TABLE 1:** REGRESSION ANALYSIS OF THE CALIBRATION CURVES FOR RAM AND LRT

Parameters	LRT	RAM
Linearity ranges (mcg/ml)	0.04 - 100	0.2 - 300
Slope	98834	41657
Standard deviation of slope	48.383	69.714
Intercept	25877	68021
Standard deviation of intercept	51.216	28.439
Correlation coefficient (r)	0.9993	0.9994

LRT is losartan potassium; RAM is ramipril

**TABLE 2:** SUMMARY OF VALIDATION PARAMETERS FOR THE PROPOSED METHOD

Parameters	LRT	RAM
LOD (μg/ml)	0.071	0.11
LOQ (μg/ml)	0.215	0.332
Accuracy (%)	99.08-99.41	99.52-99.86
Precision (RSD^a^, %)		
Intra day (n=3)	0.45-0.56	0.43-0.78
Inter day (n=3)	0.61-0.82	0.56-0.98
Resolution	1.41	1.41
Capacity factor	3.42	4.54
Theoretical plates	30271	7877
Tailing factor	1.09	1.05
HETP	5.6×10^−5^	4.7×10^−5^
Assymmetry	1.67	0.546

RSD^a^ indicates relative standard deviation; LRT is losartan potassium; RAM is ramipril

**TABLE 3:** ASSAY RESULTS OF COMBINED DOSAGE FORM USING PROPOSED METHOD

Formulation	Labeled amount (mg)		Amount obtained (mg)^b^		%Recovery^b^	
			
	LRT	RAM	LRT	RAM	LRT	RAM
A	50	5	51.00±0.4698	5.05±0.0915	102.0± 0.156	101.03±1.83

b Mean value±standard deviation of three determinations; Tablet A is Loram-5, Unichem Ltd., containing 5 mg of ramipril (RAM) and 50 mg of losartan potassium (LRT).

Thus in proposed study, RP-HPLC method has been developed for determination of RAM and LRT in combined dosage forms. The method was validated and found to be simple, sensitive, accurate and precise. The method was successfully applied for determination of drugs in their pharmaceutical formulations and hence the proposed method can be used for routine analysis of RAM and LRT in combined dosage form.
